# Leveraging pretrained language models for seizure frequency extraction from epilepsy evaluation reports

**DOI:** 10.1038/s41746-025-01592-4

**Published:** 2025-04-14

**Authors:** Rashmie Abeysinghe, Shiqiang Tao, Samden D. Lhatoo, Guo-Qiang Zhang, Licong Cui

**Affiliations:** 1 Department of Neurology, McGovern Medical School, The University of Texas Health Science Center at Houston, Houston, TX USA; 2 Texas Institute for Restorative Neurotechnologies, The University of Texas Health Science Center at Houston, Houston, TX USA; 3 McWilliams School of Biomedical Informatics, The University of Texas Health Science Center at Houston, Houston, TX USA

**Keywords:** Predictive markers, Epilepsy, Risk factors

## Abstract

Seizure frequency is essential for evaluating epilepsy treatment, ensuring patient safety, and reducing risk for Sudden Unexpected Death in Epilepsy. As this information is often described in clinical narratives, this study presents an approach to extracting structured seizure frequency details from such unstructured text. We investigated two tasks: (1) extracting phrases describing seizure frequency, and (2) extracting seizure frequency attributes. For both tasks, we fine-tuned three BERT-based models (bert-large-cased, biobert-large-cased, and Bio_ClinicalBERT), as well as three generative large language models (GPT-4, GPT-3.5 Turbo, and Llama-2-70b-hf). The final structured output integrated the results from both tasks. GPT-4 attained the best performance across all tasks with precision, recall, and F1-score of 86.61%, 85.04%, and 85.79% respectively for frequency phrase extraction; 90.23%, 93.51%, and 91.84% for seizure frequency attribute extraction; and 86.64%, 85.06%, and 85.82% for the final structured output. These findings highlight the potential of fine-tuned generative models in extractive tasks from limited text strings.

## Introduction

Epilepsy affects more than 3.4 million people in the United States and 65 million globally. Even with the availability of various treatments, 4 out of 10 individuals with epilepsy will continue to have uncontrolled seizures (https://www.epilepsy.com/stories/number-people-epilepsy-united-states-all-time-high-cdc-reports). Sudden Unexpected Death in Epilepsy (SUDEP) refers to the sudden and unexpected death of someone with epilepsy who was otherwise healthy, where no other cause of death can be found during an autopsy^1^. SUDEP is the leading cause of death in patients having uncontrolled seizures. Annually, more than 1 in 1000 epilepsy patients die from SUDEP (https://www.cdc.gov/epilepsy/sudep/index.html#:~:text=Causes%20of%20SUDEP&text=Heart%20problems%E2%80%94a%20seizure%20may,may%20also%20contribute%20to%20SUDEP). However, mechanisms behind SUDEP remain an active area of research^2–4^.

A number of risk factors have been identified for SUDEP^5–9^. For instance, SUDEP-7 Inventory comprises seven risk factors for assessing an individual’s SUDEP risk^10^. Notably, four of these risk factors are associated with seizure frequency, such as “More than three tonic-clonic seizures in last year,” “One or more tonic-clonic seizures in last year,” and “One or more seizures of any type over the last 12 months.” Other sources such as the Center for Disease Control (CDC) also recognize uncontrolled or frequent seizures as one of the main risk factors for SUDEP (https://www.cdc.gov/epilepsy/sudep/index.html#:~:text=Causes%20of%20SUDEP&text=Heart%20problems%E2%80%94a%20seizure%20may,may%20also%20contribute%20to%20SUDEP). Therefore, it is imperative to track seizure frequencies of epilepsy patients to inform care planning and minimize SUDEP risk.

The Center for SUDEP Research (CSR), funded by the National Institute of Neurological Disorders and Stroke, has collected clinical data from over 2,700 epilepsy patients across seven institutions in the United States and Europe, to better understand risk factors and brain mechanisms of SUDEP. The CSR dataset encompasses a range of modalities, including evaluation reports from epilepsy monitoring units (EMUs) and electrophysiological signals^11–13^. The EMU evaluation reports often contain a section detailing seizure frequency. However, this information is often presented in a narrative format (or free-text), posing challenges for automated SUDEP risk assessment. These narratives vary widely in terms of their content, with some segments containing single or multiple seizure frequencies, while others lacking any explicitly defined seizure frequency information. Therefore, there is a pressing need to extract structured seizure frequency data from EMU evaluation reports automatically to assist in assessing individual SUDEP risk for epilepsy patients.

Although natural language processing (NLP) techniques have been employed in the epilepsy domain for patient identification, risk stratification, and prediction^14^, approaches to extracting seizure frequencies from clinical text are relatively rare. In one such work, Decker et al. developed a rule-based NLP pipeline to extract seizure types and frequencies from clinical notes^15^. Their algorithm leveraged pattern matching and regular expressions to scan a given note for seizure frequency phrases and extract the seizure event type and the quantitative frequency. Xie et al. explored the fine-tuning of pre-trained Bidirectional Encoder Representations from Transformers (BERT)-based models to classify seizure freedom, extract seizure frequency, and extract the date of the last seizure^16^. They framed the task of seizure frequency extraction as an extractive question-answering task. However, the potential of employing more recent generative large language models (LLMs) for extracting structured seizure frequencies from clinical text remains to be explored.

The goal of this work is to develop an automated approach for seizure frequency extraction from EMU evaluation reports to facilitate SUDEP risk assessment. To this end, we investigated two specific tasks: (1) extracting phrases describing seizure frequency; and (2) extracting detailed seizure frequency attributes such as seizure events and quantities. We fine-tuned and compared different pre-trained language models, including BERT-based models and generative LLMs for both tasks. Combining the results from these tasks produces a structured representation of seizure frequency information. To the best of our knowledge, this is the first study to explore the potential of LLMs for extracting structured seizure frequency details from clinical text.

## Results

We conducted our experiments on a CentOS Linux 7 server equipped with 8 NVIDIA A100 GPUs, each with a memory capacity of 80GB for all models except GPT-3.5 Turbo and GPT-4. For the GPT models, we utilized the Microsoft Azure OpenAI Service (https://azure.microsoft.com/en-us/products/ai-services/openai-service). For fine-tuning BERT-based models, we performed 100 hyperparameter tuning trials utilizing Optuna, a tool for automatic hyperparameter optimization^17^. The final hyperparameters for all the models can be found in Supplementary Information (Supplementary Tables 1–5).

### Model performance for seizure frequency phrase extraction

Table 1 presents the performance of various fine-tuned models for seizure frequency phrase extraction on the test set in terms of precision, recall, and F1-score. Among the six fine-tuned models, the GPT-4 model achieved the highest mean precision (86.61%) and mean recall (85.04%), outperforming the other models in terms of mean F1-score (85.79%). This higher mean F1-score of GPT-4 compared to Llama-2-70b-hf, which obtained the second highest F1-score, was found to be statistically significant.

**Table 1:** Performance of various fine-tuned models for seizure frequency phrase extraction on the test set

Model	Precision (%)	Recall (%)	F1-score (%)
bert-large-cased	77.33 ± 4.24	71.95 ± 4.65	74.51 ± 4.2
biobert-large-cased	78.83 ± 4.06	75.43 ± 4.27	77.06 ± 3.86
Bio_ClinicalBERT	70.12 ± 4.79	65.8 ± 4.58	67.84 ± 4.29
Llama-2-70b-hf	80.72 ± 4.16	80.69 ± 3.65	80.68 ± 3.58
GPT-3.5 Turbo	84.53 ± 3.85	77.13 ± 4.15	80.64 ± 3.81
GPT-4	**86.61 ± 4.28**	**85.04 ± 3.51**	**85.79 ± 3.59**

Data are shown as mean ± standard deviation. Highest scores are highlighted.

### Model performance for seizure frequency attribute extraction

Table 2 shows the performance metrics of the six fine-tuned models for the seizure frequency attribute extraction task. Among these models, GPT-4 yielded the highest mean precision (90.23%) and mean recall (93.51%), outperforming other models in terms of mean F1-score (91.84%). The higher mean F1-score of GPT-4 compared with bert-large-cased which obtained the second highest F1-score, was found to be statistically significant.

**Table 2:** Performance of different fine-tuned models for seizure frequency attribute extraction on the test set

Model	Precision (%)	Recall (%)	F1-score (%)
bert-large-cased	87.19 ± 1.74	90.9 ± 1.35	89 ± 1.43
biobert-large-cased	87.45 ± 1.68	90.28 ± 1.34	88.84 ± 1.38
Bio_ClinicalBERT	83.98 ± 1.97	88.05 ± 1.56	85.96 ± 1.67
Llama-2-70b-hf	84.64 ± 2.68	85.83 ± 2.17	85.23 ± 2.33
GPT-3.5 Turbo	88.99 ± 1.62	90.23 ± 1.7	87.91 ± 1.61
GPT-4	**90.23** ± **1.7**	**93.51** ± **1.21**	**91.84** ± **1.36**

Data are shown as mean ± standard deviation. Highest scores are highlighted.

### Model performance for structured seizure frequency extraction

Table 3 provides a pairwise comparison of various model combinations for seizure frequency phrase extraction and attribute extraction to obtain structured seizure frequency details, with all scores reported as F1-scores. The results indicate that using GPT-4 for both seizure frequency phrase and attribute extraction yielded the highest performance for structured seizure frequency extraction, with a mean F1-score of 85.82% (mean precision: 86.64%, mean recall: 85.06%). The combination of GPT-3.5 Turbo for seizure frequency phrase extraction and GPT-4 for seizure frequency attribute exaction performed similarly with a mean F1-score of 85.25%. The performance difference between the former and the latter was not found to be statistically significant.

**Table 3:** Pairwise comparison of model combinations for extracting structured seizure frequency details

Attributes	Phrase	bert-large-cased	biobert-large-cased	Bio_ClinicalBERT	Llama-2-70b-hf	GPT-3.5 Turbo	GPT-4
bert-large-cased		73.34 ± 4.32	79.6% ± 3.59	69.67 ± 4.15	80.68 ± 3.26	81.62 ± 3.65	83.17 ± 3.67
biobert-large-cased		71.49 ± 4.24	75.04 ± 3.85	69.4 ± 4.18	78.06 ± 3.63	77.03 ± 3.91	80.52 ± 3.85
Bio_ClinicalBERT		68.44 ± 4.5	76.86 ± 3.78	66.91 ± 4.18	78.05 ± 3.42	78.86 ± 3.84	78.73 ± 3.91
Llama-2-70b-hf		70.59 ± 4.44	78.13 ± 3.82	67.83 ± 4.22	81.04 ± 3.31	80.7 ± 3.73	83.15 ± 3.72
GPT-3.5 Turbo		70.93 ± 4.17	78.13 ± 3.72	69.72 ± 4.1	81.05 ± 3.47	82.52 ± 3.63	83.53 ± 3.73
GPT-4		74.84 ± 4.12	77.78 ± 3.75	70.01 ± 4.06	81.93 ± 3.26	85.25 ± 3.39	**85.82** ± **3.56**

The scores listed are all F1-scores (%). Data are shown as mean ± standard deviation. The highest scores are highlighted.

## Discussion

In this paper, we investigated approaches to extract structured seizure frequency information from selected small portions of unstructured clinical text. The primary objective of this work was to facilitate accurate SUDEP risk assessment. Given EMU reports with segments like those analyzed in this work continue to be generated, our approach provides a crucial step toward automating the extraction of seizure frequency information to facilitate SUDEP risk assessment. However, the implications of this work extend far beyond SUDEP risk stratification, providing a broader impact on capturing seizure outcomes in clinical settings, including evaluating treatment efficacy and planning disease progression. By automating the extraction of seizure frequencies, our approach can contribute to more standardized and scalable methods for tracking seizure patterns, evaluating treatment efficacy, disease progression, and planning of care. Since the EMU reports used in this work are generated by a bespoke Electronic Health Record (EHR) system^18^, a tool based on this work could be integrated into the system, allowing clinicians to view and confirm structured seizure frequencies in real-time as they document the notes.

To achieve our objectives, we explored various pre-trained models to extract seizure frequency phrases and seizure frequency attributes from free-text. We aimed to integrate the output of such phrase extraction and attribute extraction models to come up with structured seizure frequencies. Until recently, sequence labeling approaches based on encoder-only models such as BERT and its derivatives were the go-to models for such natural language understanding (NLU) tasks^19^. Our aim in this work was not only to come up with an effective method for obtaining structured seizure frequencies to facilitate the SUDEP risk assessment of epilepsy patients, but also to provide a comparison of the performance of a number of popular BERT-based models and more recent generative models. Interestingly, our results revealed that GPT-4 outperforms all BERT-based models as well as Llama-2 and GPT-3.5 Turbo for both seizure frequency phrase and attribute extraction. However, the bert-large-cased model came in second for seizure frequency attribute extraction, while Llama-2 and GPT-3.5 Turbo followed GPT-4 for seizure frequency phrase extraction.

Building on these findings, we also evaluated GPT-4’s ability to handle instances lacking explicitly defined seizure frequency information. When GPT-4 was applied to the test set, it identified 104 instances as lacking explicitly defined seizure frequency information, 98 of which were correct (precision of 94.23%). On the other hand, the test set contained 99 instances lacking explicitly defined seizure frequency information, from which the model correctly identified 98 (a recall of 98.99%). This resulted in an F1-score of 96.55% for correctly detecting instances lacking explicitly defined seizure frequency information.

In this work, the structured seizure frequencies were obtained by integrating the individual outputs from phrase extraction and attribute extraction models. An alternative approach is to extract frequency phrases first and then perform attribute extraction within these phrases. Testing this approach with GPT-4 resulted in a mean precision of 84.9% ± 4.47, a mean recall of 83.36% ± 3.75, and a mean F1-score of 84.1% ± 3.84. Though this F1-score is lower than the model with the highest F1-score for structured frequency extraction (leveraging GPT-4 for both frequency phrase and attribute extraction), their difference was not found to be statistically significant. As an example, consider the text “*automotor seizure lasting 1–2* *minutes happening up to 10 times per day. Progress to tonic-clonic seizures (once every 3–4 months)*.” This alternative approach incorrectly obtained the structured frequency [Event = “automotor seizure”, Minimum duration = “1”, Maximum duration = “2”, Quantity = “10”, Temporal unit = “day”], while our original approach obtained the correct structured frequency [Event = “automotor seizure”, Quantity = “10”, Temporal unit = “day”]. Both approaches correctly identified the other structured frequency in this text: [Event = “tonic-clonic seizures”, Quantity = “once”, Minimum duration = “3”, Maximum duration = “4”, Temporal unit = “months”].

Off-the-shelf use of modern LLMs has gained attention for their potential to perform tasks without any task-specific fine-tuning. However, our findings indicate that such approaches are not as effective in extracting structured seizure frequencies from unstructured text. We employed GPT-4o in a few-shot setting to assess its effectiveness in this task. A tailored prompt containing multiple examples covering different types of attributes used in the work was developed (see Supplementary Note 1 for the prompt). The prompt guides GPT-4o through examples to respond with structured frequencies in a given JSON-like format. We applied this approach to the test set and observed that this few-shot strategy only achieved a mean precision of 49.55% ± 5.44, a mean recall of 47.34% ± 4.91, and a mean F1-score of 48.36% ± 4.86 across 10,000 bootstrap trials. This underscores the importance as well as the necessity of fine-tuning such models for optimal performance.

The approaches discussed in this paper were developed for extracting seizure frequency information from free-text segments in a specific subsection of the EMU reports reserved for holding seizure frequency information. We performed an initial investigation into how such models would perform when presented with texts that are not specifically focused on seizure frequencies. We applied the GPT-4 frequency phrase extraction and attribute extraction models on the texts under the patient history section of 30 randomly picked EMU reports which were on average 274 words long. Among these, 9 reports contained 15 seizure frequencies. However, the preliminary results indicated that the approach only correctly identified a single structured seizure frequency while incorrectly identifying 2. We further experimented with splitting the longer passages into sentences and then applying the approach to extract structured seizure frequencies individually on these sentences. This strategy led to the model accurately extracting 13 out of the 15 seizure frequencies. However, the approach also incorrectly extracted 17 structured frequencies. Therefore, we believe that the appropriate course of action to apply the approach for general text is to retrain the models on a dataset containing such text. The same pipeline that was used in this work could be adapted with a dataset containing general text.

In order to understand how the frequency values in structured frequencies extracted by different models compare with the actual frequency values, we further performed an analysis using Mean Absolute Error (MAE). To facilitate comparison, we normalized all extracted structured frequencies to a “per day” basis. Whenever a structured frequency extracted by a model had missing data that would hinder normalization (such as missing a temporal unit), we assigned the average seizure frequency for structured frequencies across the test set as a placeholder frequency for those instances. We ran 10,000 bootstrap trials using the same bootstrap samples from the earlier performance evaluation. For each trial, we computed the Mean Absolute Error (MAE) between the frequencies extracted by the model and the gold standard annotations. The resulting mean MAEs and standard deviations across all 10,000 trials are presented in Table 4. As shown, GPT-4 when used for both seizure frequency phrase and attribute extraction produced the lowest MAE of 0.0594. In comparison, using GPT-4 for phrase extraction and bert-large-case for attribute extraction resulted in a similar MAE of 0.0693, with the difference being statistically insignificant.

**Table 4:** Mean Absolute Error (MAE) scores for seizure frequencies (in number of seizures per day), obtained using different seizure frequency phrase and attribute models

Phrase	Attributes	bert-large-cased	biobert-large-cased	Bio_ClinicalBERT	Llama-2-70b-hf	GPT-3.5 Turbo	GPT-4
bert-large-cased		0.2555 ± 0.1004	0.125 ± 0.0493	0.2941 ± 0.106	0.0724 ± 0.0203	0.1221 ± 0.0512	0.0603 ± 0.0221
biobert-large-cased		0.3062 ± 0.1097	0.2331 ± 0.0773	0.2998 ± 0.1067	0.1925 ± 0.0715	0.248 ± 0.0835	0.1704 ± 0.0711
Bio_ClinicalBERT		0.2713 ± 0.1028	0.1808 ± 0.0609	0.3038 ± 0.105	0.0919 ± 0.0222	0.129 ± 0.0512	0.0857 ± 0.0241
Llama-2-70b-hf		0.2965 ± 0.1035	0.1643 ± 0.0568	0.3408 ± 0.1084	0.1204 ± 0.036	0.1547 ± 0.0586	0.0953 ± 0.0362
GPT-3.5 Turbo		0.3545 ± 0.1197	0.2263 ± 0.0855	0.3964 ± 0.1251	0.1745 ± 0.0747	0.1793 ± 0.0762	0.162 ± 0.0747
GPT-4		0.2549 ± 0.1046	0.1741 ± 0.0617	0.2952 ± 0.1065	0.0737 ± 0.0209	0.11 ± 0.0515	**0.0594** ± **0**.**022**

Results are presented as mean ± standard deviation, with the lowest error score highlighted.

To understand and analyze the impact of the findings on SUDEP risk assessment, we computed the SUDEP-7 scores based on the structured seizure frequencies extracted by different models from the test set, as well as their gold standard manual annotation. These scores were based on the first four risk factors in the SUDEP-7 inventory, as they directly correspond to seizure frequency^10^. We also performed 10,000 bootstrap trials on the same bootstrap samples obtained earlier. In each trial, we computed the MAE between the SUDEP-7 score calculated by a model’s structured seizure frequency extraction and the gold standard annotation. The mean MAEs and standard deviations computed across 10,000 bootstrap trials are given in Table 5. As can be seen, using GPT-4 for both phrase extraction and attribute extraction provided the lowest mean MAE of 0.1101 while using GPT-4 for phrase extraction and bert-large-cased for attribute extraction performed similarly with an MAE of 0.12. Their difference was not found to be statistically significant. Note that erroneous extraction of structured seizure frequencies may not always lead to inaccurate SUDEP-7 risk scores. For example, for the string “0.5 per month,” using GPT-4 for both phrase extraction and attribute extraction correctly obtains the structured frequency [Quantity = “0.5”, Temporal unit = “month”] while using GPT-4 for phrase extraction and biobert-large cased for attribute extraction incorrectly obtains [Quantity = “5”, Temporal unit = “month”]. However, both of these only satisfy the third risk factor in the SUDEP-7 Inventory: “One or more seizures of any type over the last 12 months,” both leading to the accurate SUDEP-7 risk score of 1. It must be noted that while such incorrect extractions may not impact the analysis of SUDEP risk by SUDEP-7 inventory, as the extracted seizure frequencies are inherently wrong, they may still affect other tasks leveraging these structured frequencies, such as clinical decision-making and downstream data analysis for research.

**Table 5:** Mean absolute error (MAE) scores for SUDEP-7 risk scores were obtained using different combinations of seizure frequency phrase and attribute models

Phrase	Attributes	bert-large-cased	biobert-large-cased	Bio_ClinicalBERT	Llama-2-70b-hf	GPT-3.5 Turbo	GPT-4
bert-large-cased		0.2207 ± 0.0464	0.1798 ± 0.0436	0.2551 ± 0.0521	0.13 ± 0.0357	0.1401 ± 0.0346	0.12 ± 0.0342
biobert-large-cased		0.2607 ± 0.0517	0.2101 ± 0.0462	0.2651 ± 0.0529	0.1848 ± 0.0438	0.1805 ± 0.0407	0.1604 ± 0.0405
Bio_ClinicalBERT		0.2706 ± 0.0502	0.2049 ± 0.0454	0.2752 ± 0.0533	0.145 ± 0.0372	0.1651 ± 0.0388	0.1601 ± 0.0397
Llama-2-70b-hf		0.2506 ± 0.0506	0.1898 ± 0.0444	0.2602 ± 0.0525	0.1349 ± 0.0373	0.1452 ± 0.0364	0.1302 ± 0.037
GPT-3.5 Turbo		0.2503 ± 0.0498	0.1898 ± 0.0439	0.255 ± 0.0514	0.14 ± 0.0362	0.1551 ± 0.0377	0.145 ± 0.0377
GPT-4		0.2007 ± 0.0444	0.1848 ± 0.0438	0.2449 ± 0.0512	0.135 ± 0.0374	0.1252 ± 0.0332	**0.1101** ± **0**.**0329**

Results are presented as mean ± standard deviation, with the lowest scores highlighted.

**Fig. 1:**
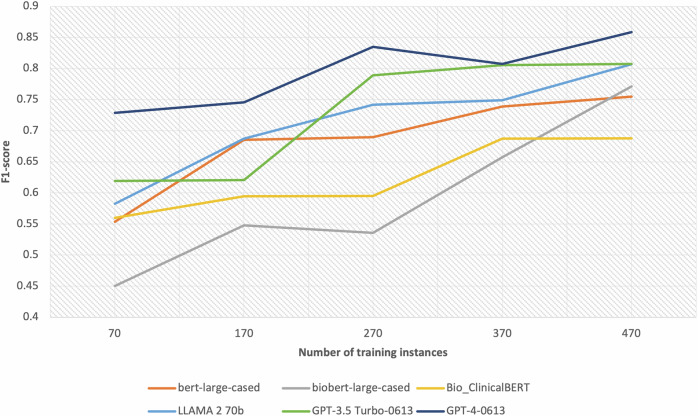
The performance of the seizure frequency phrase extraction models across different training set sizes. This figure displays the F1-scores achieved by the seizure frequency phrase extraction models as a function of varying training set sizes. It shows that GPT-4 consistently performs best across all training set sizes. Overall, all models benefitted from increased training set size.

**Fig. 2:**
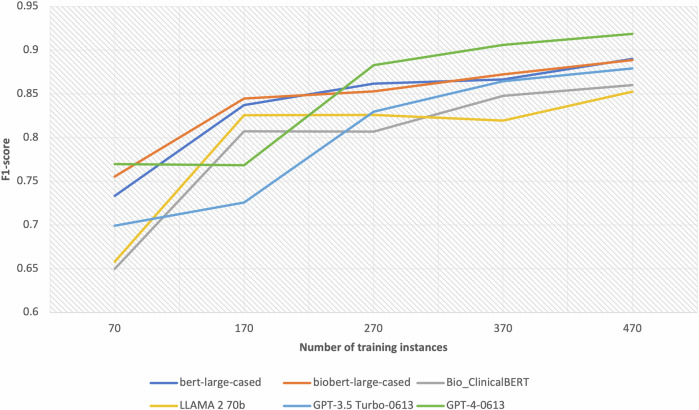
The performance of the seizure frequency attribute extraction models for different training set sizes. This figure demonstrates the F1-scores achieved by the seizure frequency attribute models as a function of different training set sizes. It shows that GPT models follow a similar trend with a notable increase in the F1-score when the training set size is increased from 170 to 270. The BERT models, together with Llama-2, also follow a similar trend with a notable increase in performance when the training dataset is increased from 70 to 170.

Our initial training set consisted of 470 instances. To understand the effect of training set size on the performance of models for extracting seizure frequency phrases and attributes, we experimented with smaller sets of 370, 270, 170, and 70 training instances, randomly chosen from the original training set. Figure 1 depicts the F1-scores for seizure frequency phrase extraction models trained on these different sizes. Both GPT models’ F1-scores took a notable drop at 170 training instances. The biobert-large-cased model, which had the 4th best performance with 470 training instances, experienced a notable decline at smaller training instances. Other models maintained reasonable performance down to 170 instances compared to their full training set performance. Figure 2 presents the F1-scores for the seizure frequency attribute extraction models across various training set sizes. Similar to the seizure frequency phrase extraction, both GPT models again showed a notable decrease in performance at 170 training instances. Other models experienced a notable drop in performance when the training set was reduced from 170 to 70 instances, although the biobert-large-cased and bert-large-cased models maintained good F1-scores at 170 instances.

There are instances where the model makes extraction errors. In some cases, the models may incorrectly identify an entity. For example, in extracting seizure frequency attributes from the text “*5 times every morning*,” GPT-4 incorrectly extracted the word “every” as a Duration. In other scenarios, the models may only partially identify an entity. For instance, in extracting seizure frequency phrases from the text “*Once daily for automotor sz and 2 per year for GTC*,” GPT-3.5 Turbo extracted “Once daily” and “2 per year” as seizure frequency phrases but missed the seizure events “automotor sz” and “GTC” respectively. The models may also fail to identify entities. For example, in extracting seizure frequency phrases from the text “*Multpkle over past two yeasr (5-10* *sec each)*”[sic], GPT-4 failed to identify a frequency phrase within this text. All such scenarios are considered incorrect extractions in our work. However, in some variations of the partial identification case above, though we consider the extracted seizure frequency to be incorrect, the structured seizure frequency extracted by combining the extracted seizure frequency with the attributes may still be accurate. Take the text “*Few per hour*.” as an example, our manual frequency phrase annotation was “Few per hour” (without the period), while GPT-4 extracted the entire text “Few per hour.” (with the period) leading this to be classified as an incorrect extraction. The GPT-4 model, however, correctly identified the attributes “few” as a Quantity and “hour” as a Temporal unit in this text. Combining the outputs of the two models, the structured frequency obtained was: [Quantity = “Few”, Temporal unit = “hour”], which is accurate. This is the reason why some structured seizure extraction models outperformed their underlying seizure frequency extraction models.

Few studies have explored NLP approaches for extracting seizure frequency information from clinical notes. Decker et al. presented a rule-based system achieving an F1-score of 0.82 on their test set^15^. Compared to their approach, our model can extract more complex scenarios involving ranges (minimums and maximums) of seizure quantities and durations, as well as additional temporal expression types such as ages, specific times, relative times, and relative time periods. Decker et al.’s results indicated that rule-based systems do not generalize well across data from different institutions, whereas our data is sourced from six different institutions. Moreover, rule-based systems require extensive time and effort to develop rules, hindering their quick adaptation to different institutional data. Xie et al.’s transfer learning approach was targeted towards classifying seizure freedom and extracting seizure frequency and date of last seizure^16^. For extracting seizure frequencies, in contrast to our token-classification approach, they explored extractive question-answering models, with the best model achieving an F1-score of 0.845. However, their approach could only extract seizure frequency phrases, necessitating additional steps to extract detailed seizure frequency attributes.

Several limitations of this study warrant discussion. One limitation is that we randomly selected segments for our dataset as well as the splits without stratifying by institution. There could be institution-specific jargon, customs, and reporting styles which may introduce slight biases impacting generalizability. Therefore, we intend to perform a comprehensive analysis of institution-specific biases in the future so that the robustness of the models against potential institution-specific biases can be identified. In addition, since EMUs often capture data primarily from individuals who may not respond to medications or are undergoing evaluation to determine an epilepsy diagnosis, our dataset may lack adequate representation of seizure-free cases and may limit the model’s generalizability to broader clinical populations. Additionally, the model was trained to extract explicit seizure frequency information; thus, if no seizure frequency information is extracted, it only reflects a lack of explicitly defined seizure frequency information and remains ambiguous regarding the status of “no seizures” or “seizure freedom.” Another limitation is that our evaluation is too rigid since it requires the starting and ending position of any entity extracted to exactly match the manual annotations to be considered an accurate extraction. In future work, we expect to investigate more flexible evaluation approaches that take into account partial matches enhancing the robustness of the evaluation and better reflecting the real-world performance of the models. The current approach requires 17 different attribute types to represent different seizure frequencies. We plan to investigate leveraging the reasoning power of LLMs to reduce some of these attributes so that a more concise set of attributes can be used, thereby reducing the harmonization and normalization efforts. For instance, an LLM would be potentially able to infer that “2003 to 2010” represents 7 years, therefore, not requiring the “Interval start” and “Interval end” attribute types.

In this work, we investigated various models for two tasks: extracting seizure frequency phrases and extracting seizure frequency attributes. We then combined their outputs to obtain structured details on seizure frequency. Although the results are promising, there is room for improvement. Currently, the models for extracting seizure frequency phrases and attributes operate independently. However, the output from one model could potentially inform the other. For example, the seizure frequency attributes of a given text may be useful in identifying its seizure frequency phrase. Therefore, we plan to investigate how the outputs of one model can be integrated into another to enhance prediction performance. In addition, for LLMs, we only investigated GPT-4, GPT-3.5 Turbo, and Llama-2 models. Our work demonstrates the potential of generative models for this text extraction task, which has traditionally been dominated by encoder-only models like BERT. Future work will involve more comprehensive comparisons to investigate if there are other generative LLMs better suited for this task.

In this paper, we presented an automated approach for extracting structured seizure frequency details from clinical text. Our approach involved tackling two key extraction tasks: seizure frequency phrase extraction and seizure frequency attribute extraction. For both tasks, we experimented with fine-tuning three pre-trained BERT models (bert-large-cased, biobert-large-cased, and Bio_ClinicalBERT), and instruction tuning three generative large language models (GPT-4, GPT-3.5 turbo, and Llama-2-70b-hf). The final structured seizure frequency details were derived by combining the outputs from both tasks. Our experiments showed that by using GPT-4 model for both seizure frequency phrase and attribute extraction achieved the best performance with a precision of 86.64%, recall of 85.06%, and F1-score of 85.82%. These results highlight the potential of generative large language models for extractive tasks.

## Methods

**Fig. 3:**
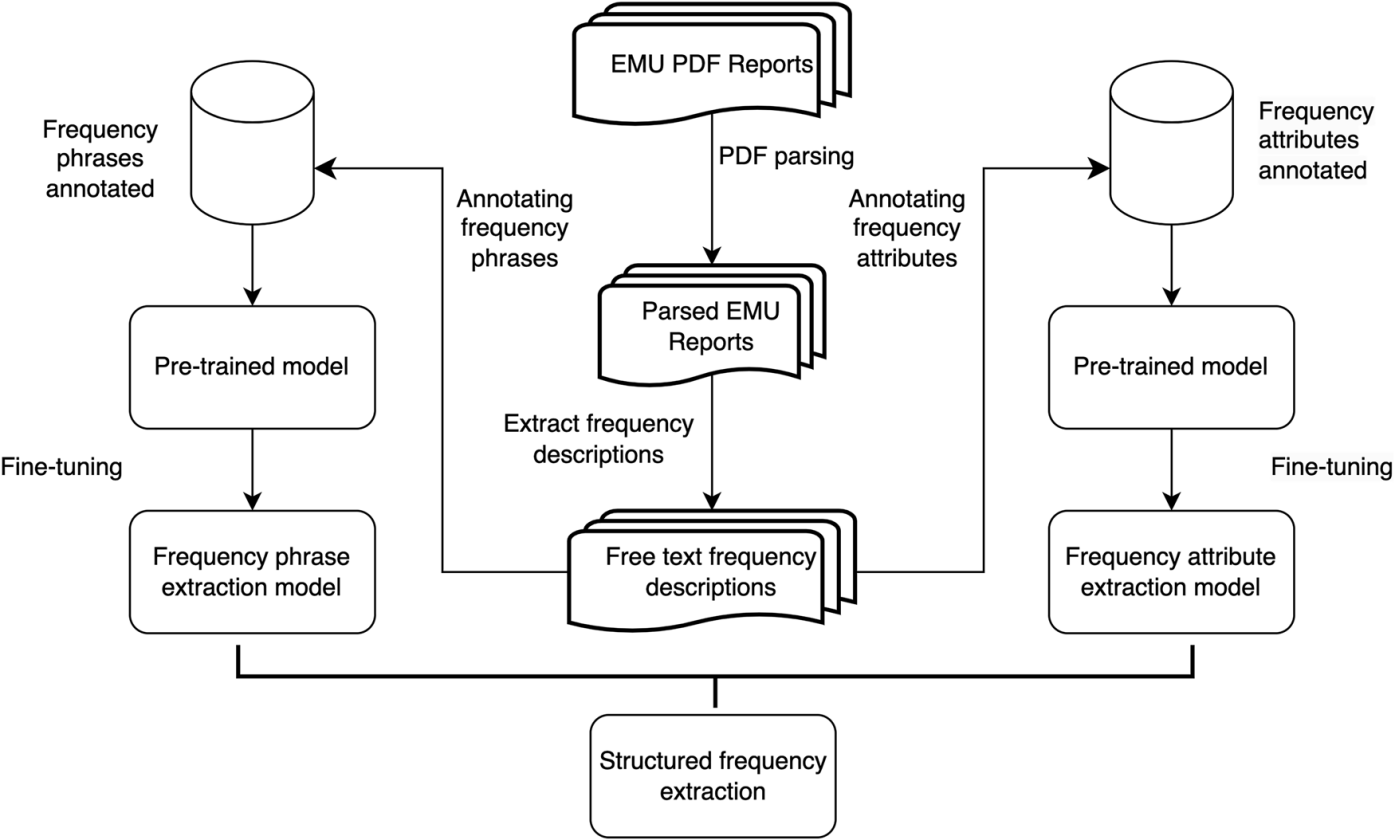
The seizure frequency extraction workflow. This figure demonstrates the workflow followed to extract structured seizure frequencies from EMU PDF reports. As shown, the PDF reports are parsed and frequency phrases and attributes are annotated. Separate pre-trained models are fine-tuned for frequency phrase extraction and attribute extraction. Their outputs are combined to obtain structured seizure frequencies.

In this work, we utilized EMU reports in PDF format from the CSR dataset to develop an automated method for seizure frequency extraction. Our approach involves two main tasks: (1) extraction of seizure frequency phrases; and (2) extraction of detailed seizure frequency attributes. For each task, we fine-tuned different pre-trained language models including three BERT-based models and three generative LLMs. By merging the output of the two tasks, we can systematically extract structured seizure frequency details for each instance mentioned in the text. Figure 3 shows the overall workflow of our approach.

### Dataset preparation

In this work, we utilized EMU reports in PDF format from the CSR dataset, which were parsed and converted into plain text files with the pdftotext tool (https://www.xpdfreader.com/pdftotext-man.html). The EMU reports contain a subsection reserved for holding seizure frequency information. From these subsections, we extracted 2242 free-text segments through a Python script. These were found to be originating from 6 different institutions. To train and evaluate various models, we randomly selected 800 instances as our dataset for manual annotation. Table 6 presents a summary of characteristics of our dataset, including the number of free-text segments based on originating institutions, statistics about the number of words in these free-text segments, and the number of seizure frequencies in the free-text segments. Note that out of the 800 segments, 377 contained one frequency, 39 contained two frequencies, 2 segments contained three frequencies, 1 segment contained four frequencies, and 381 did not contain explicitly defined seizure frequency information.

**Table 6:** Summary of characteristics of the dataset

Number of free-text segments from different institutions	University Hospitals Cleveland Medical Center	334
	Northwestern University	194
	University of Iowa	86
	University College London	78
	Thomas Jefferson University	56
	New York University	52
Number of words in free-text segments	Mean	8.72
	Median	6
	Minimum	1
	Maximum	54
Number of seizure frequencies in free-text segments	Mean	0.58
	Median	1
	Minimum	0
	Maximum	4

The seizure frequency phrases and detailed attributes in these 800 instances were manually annotated by authors RA and LC (who have prior experience in information extraction from biomedical text) using the open-source annotation tool Doccano (https://github.com/doccano/doccano). For example, the bolded phrases in the following description are mentions of two distinct seizure frequencies:

“**Aura- 1–2 per week** (unclear if it is related to sensation of muscle contraction at onset of clonic seizure), **GTC 1 every 5–6 months**”

As can be seen from the above example, a seizure frequency phrase may involve various types of attributes such as seizure event, quantity, duration, and temporal unit. Table 7 shows a list of all the attribute types used in manual annotation, alongside example phrases illustrating each attribute type.

**Table 7:** Attribute types used for annotating seizure frequency phrases and illustrating examples

Attribute type	Example
Event	3 **aphasic seizures** per week and 1 **GTC seizure** every 6 months
Quantity	**3** aphasic seizures per week and **1** GTC seizure every 6 months
Temporal unit	3 aphasic seizures per **week** and 1 GTC seizure every 6 **months**
Minimum quantity	Aura- **1**–2 per week
Maximum quantity	Aura- **1**–**2** per week
Interval start	5 seizures from **2003** to 2013
Interval end	5 seizures from 2003 to **2013**
Time	total of 3 events in the month of **June, 2018**
Relative time	Last known **2 years ago**
Relative time period	Once per month in the **last 10 months**
Duration	3 every **4** months
Minimum duration	one to two seizures every 4–5 months
Maximum duration dduration	one to two seizures every 4–5 months
Age	Started at **7 months of age**
Age start	started at **7** **y/o**, stopped around age 8
Age end	started at 7 y/o, stopped around **age 8**
Periodic	**Daily** since 2012

The attribute values are in bold and underlined.

This dataset of 800 instances was further split into three sets: 400 for training, 200 for validation, and the remaining 200 for testing. We observed that some attribute types (*Interval start, Relative time period, Age, Age start, and Age end*) had few instances in the training set. For such cases, we employed a text augmentation strategy leveraging ChatGPT to generate additional training instances based on the existing instances (https://chatgpt.com/). The prompt that we engineered for this is given as follows:

“*Working* as *a clinical text augmentation tool that generates additional training instances for training an NLP model, provide <*the number of augmented instances needed*> augmented text for the input clinical text. The augmented text should be approximately similar in length to the input. The numerical values and units in the text must be augmented as well. Input: “<*the input training instance > “.”

### Seizure frequency phrase and attribute extraction models

The task of seizure frequency phrase extraction involves the automatic detection and extraction of phrases describing seizure frequencies within the given text. Our dataset may include three types of text: (1) text with a single seizure frequency phrase, (2) text with multiple seizure frequency phrases, and (3) text lacking explicitly defined seizure frequency information. For example, the text “*Aura- 1–2 per week*” contains a single seizure frequency and the text “*Right arm clonic 1 every 2 weeks, GTC one every 2 months*” contains two seizure frequency phrases. The texts lacking explicitly defined seizure frequency information include instances with missing temporal context (e.g., “*Happened only once*”), cases where seizure frequency cannot be clearly determined (e.g., “*Uncertain*”; “*Variable*”; or “*Patient was unable to tell the frequency, she said they became less after AEDs were started*”), and instances indicating seizure remission (e.g., “*No seizures in the last 20 years*”).

The task of seizure frequency attribute extraction is to identify specific details of a seizure frequency, such as the seizure event, its quantity, and the temporal unit (see Table 1 for a comprehensive list of potential components). For instance, the text “*1 GTC seizure every 6 months*” mentions a seizure event “GTC seizure,” a quantity of “1,” a duration of “6,” and a temporal unit of “months.”

For both seizure frequency phrase extraction and attribute extraction tasks, we investigated three pre-trained BERT models and three pre-trained generative LLMs. Despite being fine-tuned on different data, both tasks shared a common pipeline for identifying text spans (start and end positions).

To begin, we examined BERT, which is a popular pre-trained encoder-only language model developed by Google that has revolutionized Natural Language Processing^20^. Various models based on the BERT architecture have since been developed. We experimented with three BERT-based models: *bert-large-cased* (https://huggingface.co/google-bert/bert-large-cased), *biobert-large-cased*^21^ (https://huggingface.co/dmis-lab/biobert-large-cased-v1.1)*, and Bio_ClinicalBERT* (https://huggingface.co/emilyalsentzer/Bio_ClinicalBERT) for a token-classification task, aimed at assigning a label to each token in a given text (https://huggingface.co/tasks/token-classification). To accomplish this, we converted the original manual annotations of training instances to token-level tags using the IOB2 tagging scheme^22^, which enabled the assignment of a specific role to each token within a sequence. For instance, in the text “*Right arm clonic 1 every 2 weeks, GTC one every 2 months*,” the tokens “Right,” “arm,” and “clonic” were assigned IOB2 tags of B-Event, I-Event, and I-Event, respectively. This indicates that “Right” marks the beginning token of the seizure event “*Right arm clonic*,” while “arm” and “clonic” denote inside tokens. The models were then fine-tuned to predict the IOB2 tags for each token, which would ultimately be converted back to start and end string positions.

Next, we focused on three generative LLMs: Llama-2-70b-hf (https://llama.meta.com/llama2/), GPT-3.5 Turbo, and GPT-4 (https://platform.openai.com/docs/models), to explore a different strategy for extracting seizure frequency phrases and attributes. This strategy involves fine-tuning the models to generate HTML-like tags surrounding the relevant entities. The training instances were accordingly converted to a tagged format. To illustrate, the text “*Right arm clonic 1 every 2 weeks, GTC one every 2 months*” was converted to

“<FREQ>Right arm clonic 1 every 2 weeks<\FREQ>,<FREQ>GTC one every 2 months<\FREQ>”

for the purpose of extracting seizure frequency phrases. While for frequency attribute extraction, the text was converted to

“*<*EVNT*>Right arm clonic*<\EVNT><QNT>*1*<\QNT> *every*<DUR>*2*<\DUR><UNT>*weeks*<\UNT>,<EVNT>*GTC*<\EVNT><QNT>*one*<\QNT> *every*<DUR>*2*<\DUR><UNT>*months*<\UNT>.”

Here EVNT, QNT, DUR, and UNT represent the seizure attribute types Event, Quantity, Duration, and Temporal unit, respectively. As can be seen, each entity is surrounded by an opening tag (e.g., <EVNT>) and a closing tag (e.g., <\EVNT>) to indicate the starting and ending positions.

**Fig. 4:**
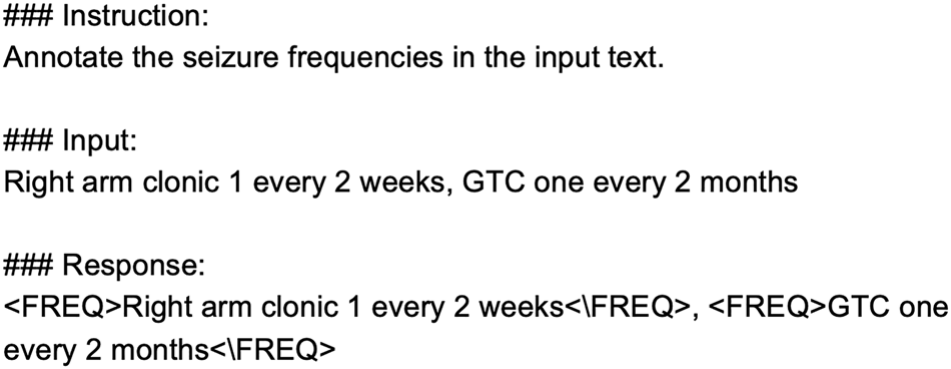
The Llama-2 input template for seizure frequency phrase extraction. This figure shows the input template used to fine-tune Llama-2 for seizure frequency phrase extraction. The “Instruction” introduces the task while the “Input” contains the segment. The “Response” contains the expected output. The “Response” is left empty at the prediction stage when the fine-tuned model is used to identify frequency phrases.

We fine-tuned Llama-2 using instruction tuning, which involves additional training of a generative LLM with a dataset of instructions and the desired outputs^23^. This supervised approach ensures the fine-tuned model produces annotations in a customized tagged format. For the seizure frequency phrase extraction, each instance of the training set was converted to the template shown in Fig. 4. For the detailed seizure frequency attribute extraction, the same template was used, with the “Response” section containing the attribute annotations. When applying the fine-tuned model to the validation set and testing set, the template remained the same, but the “Response” section was left blank. For instruction tuning, we leveraged a technique called Parameter-Efficient Fine-Tuning (PEFT) that requires fine-tuning a smaller number of parameters, greatly reducing the computational cost associated with fine-tuning the entire model^24^.

**Fig. 5:**
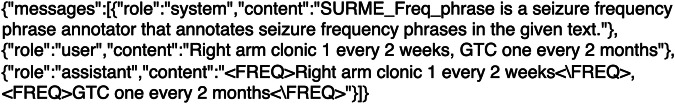
The GPT input template for seizure frequency phrase extraction. This figure shows the input template used to fine-tune GPT models for seizure frequency phrase extraction. The “system” role provides a context for the task at hand while “user” role contains the segment. The expected output will be provided in the “assistant” role. When using a fine-tuned model to extract seizure frequency phrases, the “assistant” role will not be included.

**Fig. 6:**
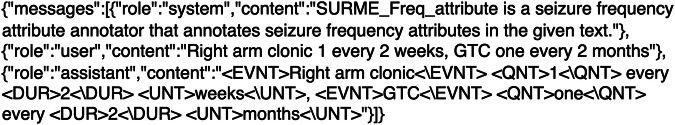
The GPT input template for seizure frequency attribute extraction. This figure displays the input template used to fine-tune GPT models for seizure frequency attribute extraction. The “system” role provides a context for the task, while the “user” role includes the segment. The expected output will be provided in the “assistant” role. When using a fine-tuned model to extract seizure frequency attributes, the “assistant” role will not be included.

For GPT-3.5 Turbo (0613 snapshot) and GPT-4 (0613 snapshot), we explored fine-tuning them through the Microsoft Azure OpenAI Service. Each training instance was formatted according to OpenAI’s requirements (https://learn.microsoft.com/en-us/azure/ai-services/openai/how-to/fine-tuning). Figure 5 and 6 show example inputs for seizure frequency phrase extraction and attribute extraction, respectively. When utilizing the fine-tuned model to extract frequency phrases or attributes from the validation set and testing set, the same template was used without the response by the role “assistant.”

Note that since Llama-2 and both GPT models produced their output in the tagged format, further post-processing was needed to pinpoint the start and end positions within the original text.

### Structured seizure frequency extraction

Since the pre-trained models were independently fine-tuned for extracting seizure frequency phrases and attributes, we investigated pairwise model combinations to obtain structured seizure frequency details. Given a seizure frequency phrase extraction model and a seizure frequency attribute extraction model, the final output of structured seizure frequency details for an input text was obtained as follows. Let the output of the seizure frequency phrase extraction model be *P* = {(*p*_*i,s*_*, p*_*i,e*_) | *0* ≤ *i* ≤ *m* and *p*_*i,e*_ < *p*_*i+1,s*_}, where *p*_*i,s*_ and *p*_*i,e*_ represent the start and end positions of the *i*-th seizure frequency phrase. Similarly, let the output of the seizure frequency attribute extraction model be *A* = {(*a*_*j,s*_*, a*_*j,e*_) | *0* ≤ *j* ≤ *n* and *a*_*j,e*_ < *a*_*j+1,s*_}, where *a*_*j,s*_ and *a*_*j,e*_ represent the start and end positions of the *j*-th seizure frequency attribute. A structured seizure frequency is defined as a subset of *A*, denoted as *S* = {(*a*_*k,s*_*, a*_*k,e*_), (*a*_*k+1,s*_*, a*_*k+1,e*_), …, (*a*_*l,s*_*, a*_*l,e*_) | *0* ≤ *k* ≤ *l* ≤ *n*}, such that there exists *(p*_*q,s*_*, p*_*q,e*_*)* in *P* such that *p*_*q,s*_ *≤ a*_*h,s*_ and *p*_*q,e*_ *≥ a*_*h,e*_ for each element *(a*_*h,s*_*, a*_*h,e*_*)* in *S*.

For example, consider the text “*Right arm clonic 1 every 2 weeks, GTC one every 2 months*.” Assume a seizure frequency phrase extraction model outputs {(0, 31), (34, 55)}, where (0, 31) represents the phrase “Right arm clonic 1 every 2 weeks” and (34, 55) represents the phrase “GTC one every 2 months.” Suppose a seizure frequency attribute extraction model outputs {(0, 15), (17,17), (25,25), (27, 31), (34, 36), (38, 40), (48,48), (50,55)} representing “Right arm clonic” (*event*), “1” (quantity), “2” (duration), “weeks” (temporal unit), “GTC” (event), “one” (quantity), “2” (duration), “months” (temporal unit), respectively. Since {(0, 15), (17,17), (25,25), (27, 31)} lies within the boundary of the phrase “Right arm clonic 1 every 2 weeks” and {(34, 36), (38, 40), (48,48), (50,55)} lies within the boundary of the phrase “GTC one every 2 months,” two structured seizure frequencies can be extracted from this example: [event = “Right arm clonic”, quantity = “1”, duration = “2”, temporal unit = “weeks”] and [event = “GTC”, quantity = “one”, duration =“2”, temporal unit = “months”].

### Performance evaluation

We evaluated the fine-tuned models on the test set, comparing their performance across different extraction tasks. The evaluation metrics included precision, recall, and F1-score. For both the seizure frequency phrase extraction and attribute extraction tasks, precision was calculated as the number of correctly extracted entities divided by the total number of extracted entities. The recall was calculated as the number of correctly extracted entities divided by the total number of manually annotated entities in the test set. An extraction was deemed correct if the span (start and end positions) exactly matched the manual annotation.

For structured seizure frequency extraction, the precision was defined as the ratio of correctly extracted structured frequencies to all extracted structured frequencies, while recall was the ratio of correctly extracted structured frequencies to the total manually annotated structured frequencies in the test set. A structured seizure frequency was considered correctly extracted only if all its seizure frequency attributes were accurately extracted.

All metrics are computed based on bootstrap trials across 10,000 bootstrapped samples from the test set, following the methods described in Yan et al. and Koehn et al.^25,26^. For each bootstrapped sample, we randomly selected 200 instances with replacements from the test set. Precision, Recall, and F1-scores are computed for each bootstrapped sample, and their mean, as well as the standard deviation, are reported. If the F1-score of one model exceeds that of another model in more than 95% of the 10,000 trials, we consider the performance of the earlier model to be statistically significantly better than the latter^25,26^.

## Supplementary information

Supplementary information

## Data Availability

The de-identified version of the text segments used in this study is available in the GitHub repository: https://github.com/rashmie/SZFreqExtract.

## Code availability

The underlying code developed for the tasks in this paper is available in the GitHub repository: https://github.com/rashmie/SZFreqExtract
