# Supplementary material for: Leveraging pretrained language models for seizure frequency extraction from epilepsy evaluation reports
Source: NPJ Digit Med. 2025 Apr 14;8:208. doi: 10.1038/s41746-025-01592-4 (PMC11997153; doi:10.1038/s41746-025-01592-4)
Supplement: Supplementary file 1 — Supplementary information [file 41746_2025_1592_MOESM1_ESM.pdf]

SUPPLEMENTARY INFORMATION: Leveraging pretrained language models for seizure frequency extraction from epilepsy evaluation reports

Rashmie Abeysinghe[1,2], Shiqiang Tao[1,2], Samden D. Lhatoo[1,2], Guo-Qiang Zhang[1,2,3], Licong Cui[2,3*]

[1]Department of Neurology, McGovern Medical School, The University of Texas Health Science Center at Houston, Houston, TX 77030, USA

[2]Texas Institute for Restorative Neurotechnologies, The University of Texas Health Science Center at Houston, Houston, TX 77030, USA

[3]McWilliams School of Biomedical Informatics, The University of Texas Health Science Center at Houston, Houston, TX 77030, USA

Table of contents

- Supplementary Table 1: Hyperparameters for the model bert-large-cased.
- Supplementary Table 2: Hyperparameters for the model dmis-lab/biobert-large-cased-v1.1
- Supplementary Table 3: Hyperparameters for the model emilyalsentzer/Bio_ClinicalBERT
- Supplementary Table 4: Hyperparameters for the model Llama 2.
- Supplementary Table 5: Hyperparameters for the models GPT-3.5 Turbo and GPT-4
- Supplementary Note 1: Prompt template used for the few-shot experiment

Supplementary Table 1: Hyperparameters for the model bert-large-cased.

Hyperparameter	Value for frequency phrase extraction	Value for frequency attribute extraction
learning_rate	0.00010962472970355563	7.292674315011053e-05
per_device_train_batch_size	8	32
num_train_epochs	3	18
weight_decay	6.175068452799282e-06	2.0591104679996593e-06

Supplementary Table 2: Hyperparameters for the model dmis-lab/biobert-large-cased-v1.1

Hyperparameter	Value for frequency phrase extraction	Value for frequency attribute extraction
learning_rate	3.732277916659287e-05	5.850448839095984e-05
per_device_train_batch_size	8	16
num_train_epochs	4	7
weight_decay	0.0019605681370994054	0.0023057979606364858

Supplementary Table 3: Hyperparameters for the model emilyalsentzer/Bio_ClinicalBERT

Hyperparameter	Value for frequency phrase extraction	Value for frequency attribute extraction
learning_rate	8.288629791776871e-05	0.00011249114894981418
per_device_train_batch_size	16	4
num_train_epochs	4	5
weight_decay	0.0027543913222117405	1.1557717651107671e-06

Supplementary Table 4: Hyperparameters for the model Llama 2.

Hyperparameter	Value for both frequency phrase and attribute extraction

lora_alpha (for PEFT)	32
lora_dropout (for PEFT)	0.01
r (for PEFT)	64
bias (for PEFT)	lora_only
task_type (for PEFT)	CAUSAL_LM
learning_rate	1e-3
per_device_train_batch_size	4
num_train_epochs	8

Supplementary Table 5: Hyperparameters for the models GPT-3.5 Turbo and GPT-4

Hyperparameter	Value for both frequency phrase and attribute extraction
n_epochs	8
batch_size	4
learning_rate_multiplier	5

Supplementary Note 1: Prompt template used for the few-shot experiment

The following is the prompt template used in the experiment to assess the effectiveness of a few-shot approach in extracting structured seizure frequencies. Note that <segment> will be replaced by the particular segment. The LLM (GPT-4o in this experiment) is guided through the examples to provide its outputs in a specific format.

Please read the following passage and extract seizure frequencies existing in the passage. Do not infer or include anything that is not explicitly mentioned in the passage. Output should be in JSON format as shown in the examples. If there are no seizure frequencies in the passage, leave the "Frequencies" array field empty. If there are multiple frequencies, multiple entries under the "Frequencies" array field should be made.

Use only the following entity types for specifying attributes of frequencies: Event, Quantity, Minimum quantity, Maximum quantity, Temporal Unit, Time, Duration start, Duration end, Relative time period, Relative time, Duration, Minimum duration, Maximum duration, Age, Age start, Age end, Periodic

Some examples are given below. Please follow the examples when coming up with your responses.

Examples:

1) Input: "Aura 3-4 per month"
Output:

```
{{
    "Frequencies":[
        {{
            "Event": "Aura",
            "Minimum quantity": "3",
            "Maximum quantity": "4",
            "Temporal Unit": "month"
        }}
    ]
}}
```

2) Input: "10 seizures from 2004 to 2010"
Output:

```
{{
    "Frequencies":[
        {{
            "Event": "seizures",
            "Quantity": "10",
            "Duration start": "2004",
            "Duration end": "2010"
        }}
    ]
}}
```

3) Input: "3 events in the month of April, 2015"
Output:

```
{{
    "Frequencies":[
        {{
            "Event": "events",
            "Quantity": "3",
            "Time": "April, 2015"
        }}
    ]
}}
```

4) Input: "Three episodes in last four months"
Output:

```
{{
    "Frequencies":[
        {{
            "Event": "episodes",
            "Quantity": "Three",
            "Relative time period": "last four months"
        }}
    ]
}}
```

5) Input: "4 every 6 months"
Output:

```
{{
    "Frequencies":[
        {{
            "Quantity": "4",
            "Duration": "6",
            "Temporal Unit": "months"
        }}
    ]
}}
```

6) Input: "four to five seizures every 3-4 months"
Output:

```
{{
    "Frequencies":[
        {{
            "Event": "seizures",
            "Minimum quantity": "four",
            "Maximum quantity": "five",
            "Minimum duration": "3",
            "Minimum duration": "4",
            "Temporal Unit": "months"
        }}
    ]
}}
```

7) Input: "30 seizures from age 6 to age 10"
Output:

```
{{
    "Frequencies":[
        {{
            "Event": "seizures",
            "Quantity": "30",
            "Age start": "age 6",
```

```
            "Age end": "age 10"
        }}
    ]
 }}
```

8) Input: "weekly seizures"
Output:

```
{{
    "Frequencies":[
        {{
            "Event": "seizures",
            "Periodic": "weekly"
        }}
    ]
 }}
```

9) Input: "20 seizures in his 30s"
Output:

```
{{
    "Frequencies":[
        {{
            "Event": "seizures",
            "Quantity": "20",
            "Age": "30s"
        }}
    ]
 }}
```

10) Input: "20 seizures in his 30s"
Output:

```
{{
    "Frequencies":[
        {{
            "Event": "seizures",
            "Quantity": "20",
            "Age": "30s"
        }}
    ]
 }}
```

11) Input: "5 aphasic seizures per month and 2 GTC seizure every 6 months"
Output:

```
{{
    "Frequencies":[
        {{
```

"Event": "aphasic seizures",
"Quantity": "5",
"Temporal Unit": "month"
}},
{{
"Event": "GTC",
"Quantity": "2",
"Duration": "6",
"Temporal Unit": "month"
}}
]
}}

12) Input: "Patient is no longer having seizures"
Output:
{{
"Frequencies":[]
}}

13) Input: "10 seizures"
Output:
{{
"Frequencies":[]
}}

Input: <segment>

Output:
